# Anomalous optical gradient force induced by polarization-tuned antisymmetry in energy density gradient

**DOI:** 10.1515/nanoph-2025-0223

**Published:** 2025-09-10

**Authors:** Lv Feng, Ziyi Su, Ruohu Zhang, Zhigang Li, Bingjue Li, Guanghao Rui

**Affiliations:** Department of Optical Engineering, School of Electronic Science and Engineering, Southeast University, Nanjing, Jiangsu, 211189, China; School of Mechanical Engineering, Southeast University, Nanjing, Jiangsu, 211189, China

**Keywords:** optical tweezers, optical gradient force, energy density gradient, polarization, electromagnetic symmetry

## Abstract

The spatial inhomogeneity of electromagnetic energy density in an optical field typically gives rise to conservative gradient forces, which serve as the fundamental mechanism for trapping nanoparticles in optical tweezers. Surprisingly, however, we demonstrate that even in the absence of an energy density gradient, optical gradient forces can still act on isotropic, achiral particles when the incident field consists of counter-propagating plane waves engineered to exhibit polarization-controlled antisymmetry between the electric and magnetic energy density gradients. Through both numerical simulations and analytical derivations based on multipole expansion theory, we show that this anomalous gradient force arises from the electromagnetic symmetry breaking induced by the particle itself, irrespective of its size. Notably, this electromagnetic symmetry breaking-induced gradient force reaches its maximum under elliptical polarization at the specific position, rather than linear or circular polarization, underscoring the critical role of polarization configuration in modulating energy density gradients. These findings reveal a previously unrecognized mechanism for optical gradient force generation and deepen our understanding of the role of hidden antisymmetry in structured light fields.

## 1 Introduction

The momentum transfer effect in light–matter interactions constitutes a fundamental physical phenomenon in the research of optical force [1], [2], [3]. Over the past decade, significant advancements in optical field manipulation technologies have propelled the study of micro- and nano-particle dynamics to new heights [4], [5], [6], [7]. Among the various manifestations of optical forces, radiation pressure emerges as the most intuitive mechanical effect, its theoretical origins tracing back to Kepler’s astronomical explanation of the directional behavior of comet tails [8]. A landmark breakthrough was achieved in 1970, when Ashkin successfully demonstrated laser trapping and manipulation of micron-sized particles through the use of two opposing beams [9]. Building on this, Ashkin further developed three-dimensional optical trapping technology using a single, tightly focused laser beam [10]. This pioneering work not only laid the physical foundation for optical tweezers but also unveiled optical gradient force as a novel and profound mechanism governing light–matter interactions.

With the rapid development of micro- and nano-photonics, the application scope of optical gradient forces has expanded beyond fundamental physics research into diverse fields [11], [12], [13], [14], [15], [16], [17], [18] such as biomedicine and materials science, enabling the precise manipulation of particles across a broad size range, from the nanoscale to the microscale, encompassing birefringent crystals [16], biological cells [17], and chiral molecules [18]. Particularly noteworthy is the emergence of long-range optical pulling forces arising from negative optical field gradients [19], as well as enantioselective trapping techniques within chiral gradient force fields [20], [21], [22], [23], [24], both of which underscore the unique advantages of spatially modulated optical fields in the manipulation of complex particles. Theoretical investigations have further revealed that the generation of chiral gradient forces is intrinsically linked to the gradient of magnetoelectric energy density [25]. In the case of achiral Rayleigh particles, the underlying force mechanism extends beyond the conventional intensity gradient (the electromagnetic energy density gradient) to incorporate the synergistic influence of the phase gradient [26], [27], [28]. Under the paraxial approximation, particular significance is the observation that the electromagnetic energy density gradient reflects the dual symmetry properties of the underlying electric and magnetic fields [29], [30]. However, early theoretical models attributed the gradient force on nonmagnetic particles solely to the electric field energy gradient [31], [32], [33], thereby confining the interpretation of local dynamic characteristics to electrical components. This restriction is now understood to stem from the dual asymmetry inherent in local light–matter interactions.

In this work, we demonstrate that an isotropic, achiral spherical particle can experience a counter-intuitive longitudinal gradient force, even when the illuminating field exhibits no net energy density gradient. This phenomenon is enabled by the polarization-tuned antisymmetry between the electric and magnetic components of the energy density gradient of the light field. Unlike conventional gradient forces that arise directly from spatial intensity variations, the force observed here originates from the symmetry breaking of the electromagnetic induced by the presence of the particle. This mechanism is revealed through a comprehensive analysis valid for the particle of arbitrary size. Remarkably, by tuning the incident angle and adjusting the polarization orientation of the incoming light, the antisymmetric distribution of electric and magnetic energy density gradients can be disrupted, generating a gradient force acting on the particle with the electromagnetic symmetry. Furthermore, the electromagnetic symmetry-breaking induced gradient force reaches its maximum under elliptical polarization at the specific position, rather than under linearly or circularly polarized light. By precisely modulating the particle radius, we isolate the respective contributions of electric and magnetic energy density gradients to the overall force, offering deeper insight into the fundamental mechanisms at play. These findings not only advance our understanding of polarization-mediated optical gradient forces but also open new avenues for applications in optical field characterization and light-driven particle manipulation.

## 2 Results and discussion

**Figure 1:**
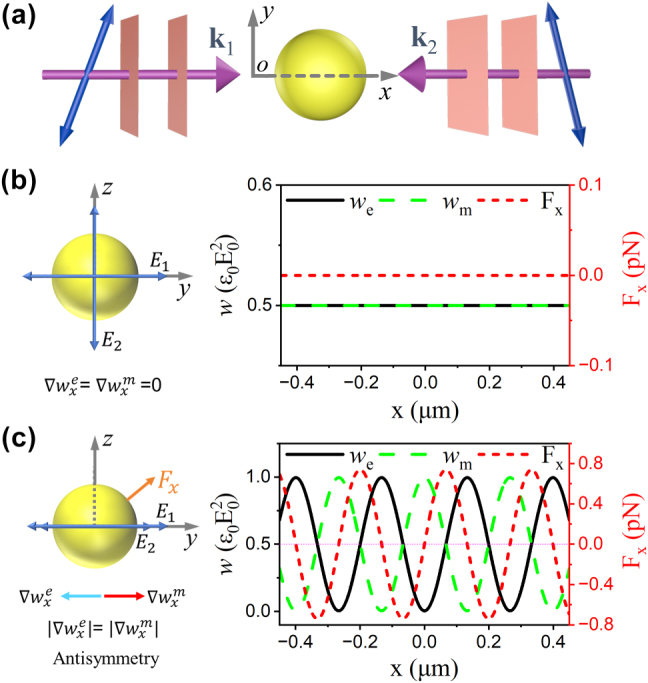
Schematic illustration of the longitudinal optical force arising from polarization-tuned antisymmetry in a two-wave interference field. (a) An achiral spherical particle is illuminated by a standing wave formed by two counter-propagating plane waves with linear polarizations. (b, c) The longitudinal optical force and the corresponding electric and magnetic energy densities as functions of the particle position, for incident fields with (b) orthogonal (y- and z-) polarizations and (c) identical (y-) polarizations. The incident wavelength is 532 nm. The particle has a radius of 400 nm and is immersed in vacuum, with a relative permittivity of 2.53 and relative permeability of 1.0.

To illustrate the anomalous gradient force, an isotropic particle is considered to be immersed in an interference field generated by two counter-propagating plane waves with identical amplitude *E*_0_ = 8.6832 × 10^5^ V/m, wavelength *λ* = 532 nm, and wave vectors oriented along the *x*-axis (as shown in Figure 1a). The resulting electric field of the interference pattern is expressed as $E=E_{0}E_{1}{\text{e}}^{\text{i}k_{1}\cdot r}+E_{0}E_{2}{\text{e}}^{\text{i}k_{2}\cdot r}$ , where $k_{j}=kcos\left(\varphi_{j}\right)\hat{x}$ denotes the wave vector of the *j*-th plane wave, *k* is the wave number in vacuum, and position vector is given by $r=x\hat{x}+y\hat{y}+z\hat{z}$ . The complex amplitude of each plane wave is described by $E_{j}=\sin\left(\alpha_{j}\right){\text{e}}^{\text{i}\beta_{j}}\cos\left(\varphi_{j}\right)\hat{y}-\cos\left(\alpha_{j}\right)\hat{z}$ where *φ*_*j*_ is the angle between the wave vector and the *x*-axis, viz. *φ*_1,2_ = 0, *π*, *α*_*j*_ describes the angle between the electric field direction and the *z*-axis, and *β*_*j*_ indicates the phase difference between the *z* and *y* components of the electric field. The energy density of optical field is expressed as [34]:

(1) $$w=\frac{1}{4}\left(\varepsilon_{0}{\left|E\right|}^{2}+\mu_{0}{\left|H\right|}^{2}\right)=w_{e}+w_{m},$$

where *ɛ*_0_ and *μ*_0_ are the permittivity and permeability in the vacuum, respectively, and *w*_*e*_ and *w*_*m*_ are the electric and magnetic parts of the energy density, given by:

(2) $$\begin{array}{c} \begin{array}{cc} w_{e} & =\frac{\varepsilon_{0}E_{0}^{2}}{2}\left[1+\cos\alpha_{1}\cos\alpha_{2}\cos\left(2kx\right)\right.\\ & \;-\left.\sin\alpha_{1}\sin\alpha_{2}\cos\left(2kx+\beta_{1}-\beta_{2}\right)\right], \end{array}\\ \begin{array}{cc} w_{m} & =\frac{\varepsilon_{0}E_{0}^{2}}{2}\left[1-\cos\alpha_{1}\cos\alpha_{2}\cos\left(2kx\right)\right.\\ & \;+\left.\sin\alpha_{1}\sin\alpha_{2}\cos\left(2kx+\beta_{1}-\beta_{2}\right)\right]. \end{array} \end{array}$$

Accordingly, the *x-*component of the electric and magnetic energy density gradients ( $\nabla w_{x}^{e}$ and $\nabla w_{x}^{m}$ ) are expressed as:

(3) $$\begin{array}{c} \begin{array}{cc} \nabla w_{x}^{e} & =E_{0}^{2}k\varepsilon_{0}\left[-\cos\alpha_{1}\cos\alpha_{2}\sin\left(2kx\right)\right.\\ & \;+\left.\sin\alpha_{1}\sin\alpha_{2}\sin\left(2kx+\beta_{1}-\beta_{2}\right)\right], \end{array}\\ \begin{array}{cc} \nabla w_{x}^{m} & =E_{0}^{2}k\;\varepsilon_{0}\left[\cos\alpha_{1}\cos\alpha_{2}\sin\left(2kx\right)\right.\\ & \;-\left.\sin\alpha_{1}\sin\alpha_{2}\sin\left(2kx+\beta_{1}-\beta_{2}\right)\right]. \end{array} \end{array}$$

It can be clearly observed that the total energy density remains constant ( $w=\varepsilon_{0}E_{0}^{2}$ ) for illumination with arbitrary polarization states, resulting in a vanishing total energy density gradient, i.e., $\nabla w_{x}=\nabla w_{x}^{e}+\nabla w_{x}^{m}=0$ . This condition can be classified into two cases. First, when the polarization directions of the two plane waves are orthogonal (i.e., *α*_1_ = *π*/2 and *α*_2_ = 0), both the electric and magnetic energy densities remain spatially uniform ( $w_{e}=w_{m}=\varepsilon_{0}E_{0}^{2}/2$ ) in Figure 1b, which leads to $\nabla w_{x}^{e}=\nabla w_{x}^{m}=0$ , and therefore the total gradient vanishes. Second, when the electric fields of the plane waves are parallel (i.e., *α*_1_ = *α*_2_ = *π*/2), the electric and magnetic energy densities exhibit opposite periodic spatial distributions, resulting in mutually antisymmetric gradients ( $\nabla w_{x}^{e}=-\nabla w_{x}^{m}$ ), and again yielding a vanishing total gradient in Figure 1c. To further investigate the consequences of such field configurations, a Mie dielectric particle (with permittivity *ɛ*_*s*_ = 2.53, permeability *μ*_*s*_ = 1, and radius *R* = 400 nm) is placed within the interference field described above, and the longitudinal (along *x*-axis) optical force acting on the particle is calculated using full-wave simulations. In the case where both *w*_*e*_ and *w*_*m*_ are spatially uniform, no optical force is exerted on the particle, as shown in Figure 1b. This phenomenon can be readily understood, given that the gradient force acting on a dielectric particle primarily arises from the spatial variation in energy density, which is absent when ∇*w*_*x*_ = 0. However, in the antisymmetric configuration where $\nabla w_{x}^{e}=-\nabla w_{x}^{m}$ , a position-dependent longitudinal optical force is found to emerge despite the total energy density gradient being zero in Figure 1c. This counterintuitive result reveals a novel method for modulating the amplitude and sign of the electric and magnetic energy density gradients, as well as interpreting optical forces by exploiting such polarization-tuned antisymmetric mechanisms.

**Figure 2:**
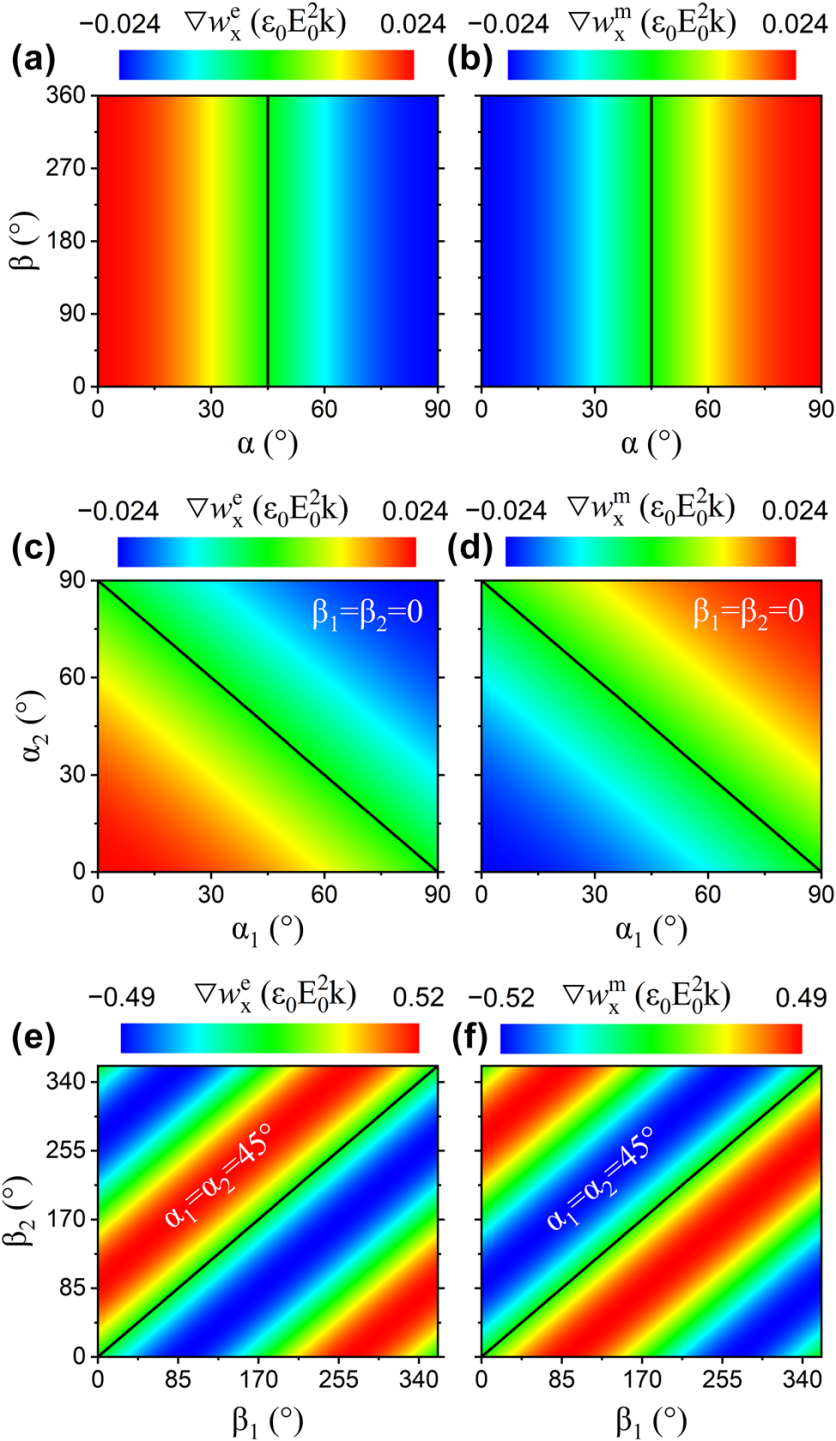
Numerical simulation of polarization-induced antisymmetry in the energy density gradient. The electric (a, c, e) and magnetic (b, d, f) energy density gradients are shown as functions of the polarization angles in two-beam interference fields under various polarization configurations. Black lines indicate conditions under which the electric or magnetic energy density gradient vanishes.

Subsequently, the influence of the polarization state of the incident field on the antisymmetric behavior is investigated at *x* = 400 nm. For two plane waves with identical polarization states (*α* = *α*_1_ = *α*_2_, *β* = *β*_1_ = *β*_2_), Figure 2a and b illustrate the variations in $\nabla w_{x}^{e}$ and $\nabla w_{x}^{m}$ as functions of polarization angle *α* and phase difference *β*. In this configuration, the gradient distribution simplifies to $\nabla w_{x}^{e}=-\nabla w_{x}^{m}=-\varepsilon_{0}kE_{0}^{2}\cos\left(2\alpha \right)\sin\left(2kx\right)$ , indicating that both the magnitude and sign of the energy density gradients are solely determined by the polarization angle *α*. The antisymmetric relation holds when *α* ≠ 45°, and $\nabla w_{x}^{e}$ ( $\nabla w_{x}^{m}$ ) reaches its maximum magnitude at *α* = 0° or 90°, corresponding to the strongest longitudinal optical force. In the case where two plane waves are linearly polarized (*β*_1_ = *β*_2_ = 0) with different polarization angles, the gradient takes the form of $\nabla w_{x}^{e}=-\nabla w_{x}^{m}=-\varepsilon_{0}kE_{0}^{2}\cos\left(\alpha_{1}+\alpha_{2}\right)\sin\left(2kx\right)$ . When the polarization angles satisfy *α*_1_ + *α*_2_ = *π*/2, interference is suppressed and both $\nabla w_{x}^{e}$ and $\nabla w_{x}^{m}$ vanish, as indicated by the black curves in Figure 2c and d. For other polarization configurations, the antisymmetric relationship remains valid. Moreover, a hidden symmetry can be identified, since both $\nabla w_{x}^{e}$ and $\nabla w_{x}^{m}$ exhibit antisymmetric distributions with respect to the black curve. For elliptically polarized plane waves with identical polarization angles (*α*_1_ = *α*_2_ = *π*/4), the gradient expression becomes $\nabla w_{x}^{e}=-\nabla w_{x}^{m}=-\varepsilon_{0}kE_{0}^{2}\left[\sin\left(2kx\right)-\sin\left(2kx+\beta_{1}-\beta_{2}\right)\right]/2$ . It is found that even when both counter-propagating waves are circularly or elliptically polarized with different states (*β*_1_ ≠ *β*_2_), the antisymmetric condition is still satisfied. Notably, the amplitude of the electric or magnetic energy density gradient is enhanced by an order of magnitude in the case of non-linearly polarized waves, compared to the linearly polarized scenario. Therefore, the antisymmetry in energy density gradients induced by polarization not only enables a highly efficient mechanism for generating longitudinal optical forces but also offers a flexible approach for modulating their magnitude and direction.

**Figure 3:**
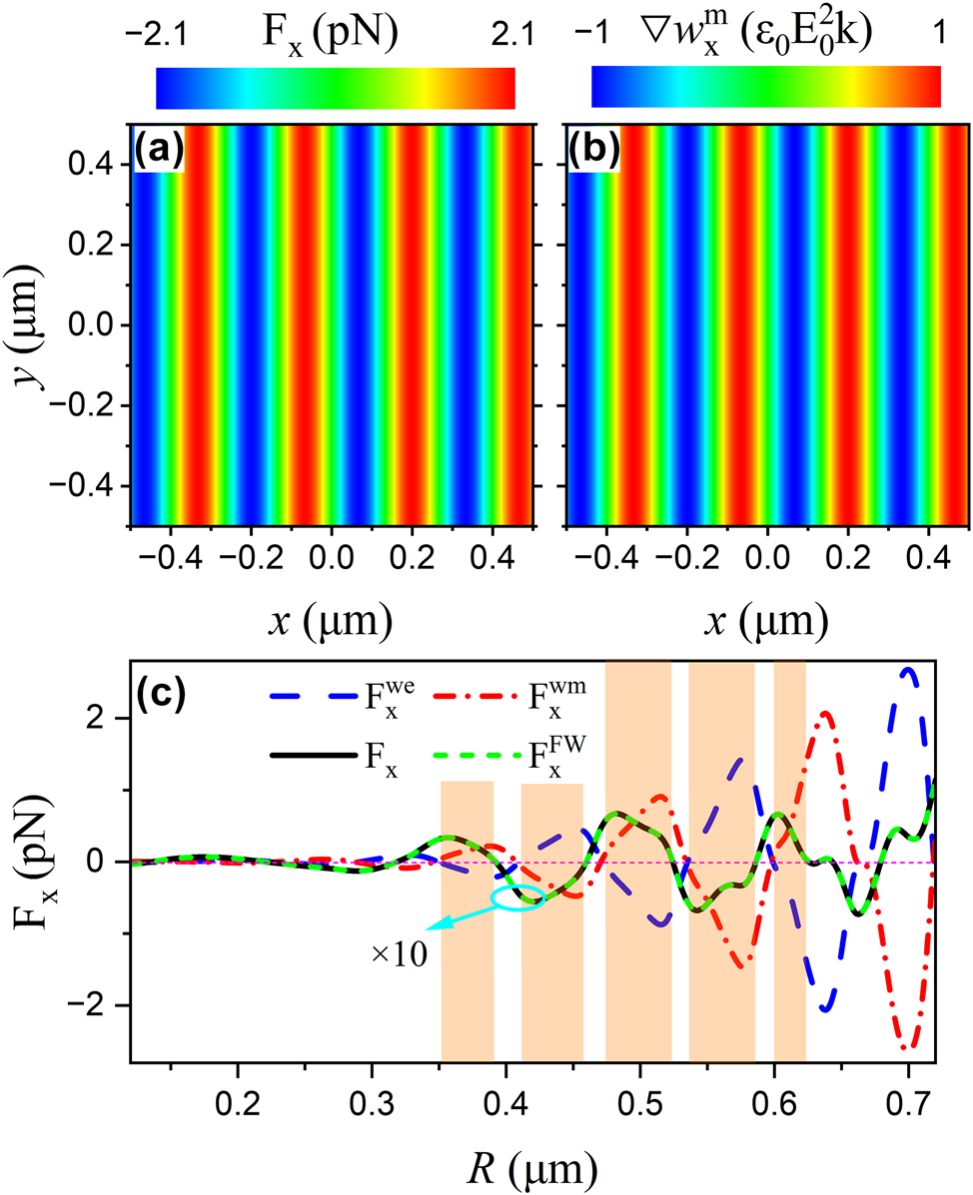
Numerical calculation of the longitudinal optical force on a polystyrene particle immersed in a two-wave interference field. (a) Spatial distribution of the optical force acting on the Mie particle with radius *R* = 500 nm. (b) Corresponding magnetic energy density gradient of the incident light field. (c) Dependence of the longitudinal optical force and its electric and magnetic energy density gradient components on the particle radius. The particle is located at *x* = 400 nm. Full-wave simulation result $F_{x}^{\text{FW}}$ (green dashed lines) is included for comparison. The notation “ × 10” indicates that the forces have been magnified by a factor of 10 to enhance visibility. The other parameters are the same as that of Figure 1c.

The full-wave simulation method was conducted based on the generalized Lorenz-Mie theory [35] and the Maxwell stress tensor method [36], enabling the determination of the spatial distribution of optical forces acting on spherical particles within the interference field. Apart from the difference in magnitude, both the optical force and the magnetic energy density gradient exhibit identical spatial periodicity, as illustrated in Figure 3a and b, thereby demonstrating a proportional relationship between the optical force and the energy density gradient. To gain an intuitive understanding of the physical mechanism underlying the emergence of longitudinal optical forces, an analytical expression is derived using multipole expansion theory [37] for the optical force acting on an achiral spherical particle under illumination by two counter-propagating plane waves (refer to Supporting Information for more details):

(4) $$\text{F}=\sum_{l=1}^{\infty }{\text{F}}_{\mathrm{int}}^{\left(l\right)}+\sum_{l=1}^{\infty }{\text{F}}_{\text{rec}}^{\left(l\right)},$$

with

(5) Fint(l)=−1l2l+1π2×Imζlal+ξlbl∇we+ξlal+ζlbl∇wm,Frec(l)=−1lχl πImγlal*al+1+ηlbl*bl+1+2υlal*bl∇we+ηlal*al+1+γlbl*bl+1−2υlal*bl∇wm,

and

(6) $$\begin{array}{c} \zeta_{l}=l^{2}+l+2,\xi_{l}=l^{2}+l-2,\\ \gamma_{l}=l^{2}+2l+3,\eta_{l}=l^{2}+2l-1,\\ \chi_{l}=\frac{l\left(l+2\right)}{l+1},\upsilon_{l}=\frac{\left(2l+1\right)}{l^{2}\left(l+2\right)}, \end{array}$$

where *l* denotes the order of electric and magnetic multipoles, and *a*_*l*_ and *b*_*l*_ represent the Mie coefficients [38]. The interception force $F_{\mathrm{int}}^{\left(l\right)}$ may be interpreted as the response of electromagnetic multipoles to incident fields, whereas the recoil force $F_{\mathrm{rec}}^{\left(l\right)}$ arises from the interplay between various multipoles excited in the particles. To simplify the formula iteration, we set *E*_0_ = *B*_0_ = *k* = *ɛ*_0_ = *μ*_0_ = 1, to derive the formula in the dimensionless case such that its optical force is in units of $\varepsilon_{b}E_{0}^{2}/k^{2}$ , where *ɛ*_*b*_ is the dielectric constant of the background. Given that both components are related to the energy density gradient, the total optical force is fundamentally a manifestation of the optical gradient force, viz. $F_{x}=F_{x}^{g}$ . Furthermore, the optical force can be decomposed into two contributions, $F_{x}^{\text{we}}$ and $F_{x}^{\text{wm}}$ , corresponding to the gradients of the electric and magnetic energy densities, respectively, i.e. $F_{x}=F_{x}^{\text{we}}+F_{x}^{\text{wm}}=\overset{\infty }{\underset{l=1}{\sum }}F_{x}^{\text{we}\left(l\right)}+\overset{\infty }{\underset{l=1}{\sum }}F_{x}^{\text{wm}\left(l\right)}$ where

(7) Fxwe(l)=(−1)l(2l+1)π2Im[ζlal+ξlbl]∇wxe+(−1)lχl πImγlal*al+1+ηlbl*bl+1+2υlal*bl∇wxe,Fxwm(l)=(−1)l(2l+1)π2Im[ξlal+ζlbl]∇wxm+(−1)lχl πImηlal*al+1+γlbl*bl+1−2υlal*bl∇wxm⋅

It becomes evident that the gradient force vanishes when both $\nabla w_{x}^{e}=0$ and $\nabla w_{x}^{m}=0$ , consistent with the observations in Figure 1b. Figure 3c presents the dependence of the longitudinal gradient force and its components on the particle radius. It can be observed that, for particles within the light orange-shaded region, the contribution from the magnetic energy density gradient surpasses that of its electric counterpart. At specific radii, the total gradient force disappears, which can be attributed to the cancellation of contributions from electric and magnetic multipoles of different orders. However, such cancellation occurs only at discrete radii and thus cannot be interpreted as arising from a universal physical mechanism. Under the specific condition where $\nabla w_{x}^{e}=-\nabla w_{x}^{m}$ , the gradient force ( $F_{x}^{g}=\overset{\infty }{\underset{l=1}{\sum }}F_{x}^{g\left(l\right)}$ ) is further simplified to:

(8) Fxg(l)= (−1)l2(2l+1)πIm[al−bl]∇wxe+(−1)l4χlπImal*al+1−bl*bl+1+υlal*bl∇wxe⋅

Most importantly, the gradient force vanishes when the Mie coefficients satisfy the relation:

(9) Imal−bl=0,Imal*al+1−bl*bl+1+υlal*bl=0.

This condition is fulfilled when *a*_*l*_ = *b*_*l*_ for all multipolar orders *l*, indicating that the presence of a particle exhibiting identical electric and magnetic responses results in the disappearance of the gradient force, irrespective of the particle’s radius. Hence, the existence of the gradient force is attributed to the breaking of electromagnetic symmetry induced by the particle. Moreover, Eqs. (5) and (8) reveal that, in addition to electromagnetic symmetry breaking, the emergence of longitudinal gradient forces necessitates a gradient in the electric or magnetic energy density, which is shown in Eq. (3) to depend on the polarization angle and the phase difference. Consequently, even when the irradiance acting on an isotropic particle does not produce a net energy density gradient along the *x*-direction, an underlying antisymmetry in the gradient field persists. This antisymmetry facilitates the coupling between particles with electromagnetic symmetry breaking and light, thereby generating anomalous longitudinal gradient forces. Additionally, as demonstrated in Figure 2, the polarization angle in Eq. (3) determines the direction of the longitudinal gradient force, which can be reversed by altering this angle. Of particular interest in this case is the observation that the magnitude of the gradient force can reach its maximum under elliptically polarized illumination, rather than with circular or linearly polarized light (refer to Figures S2 and S3 in the Supporting Information). Additionally, according to Eq (3) it is evident that the electric and magnetic energy density gradients are position dependent. The numerical results demonstrate that, within a certain spatial region, the maximum values of the electric or magnetic energy density gradient at different positions correspond to different polarization state configurations. As a result, the longitudinal gradient force can be significantly enhanced by a larger energy density gradient. This provides a controllable mechanism for tailoring the amplitude and directionality of the longitudinal force via polarization modulation.

**Figure 4:**
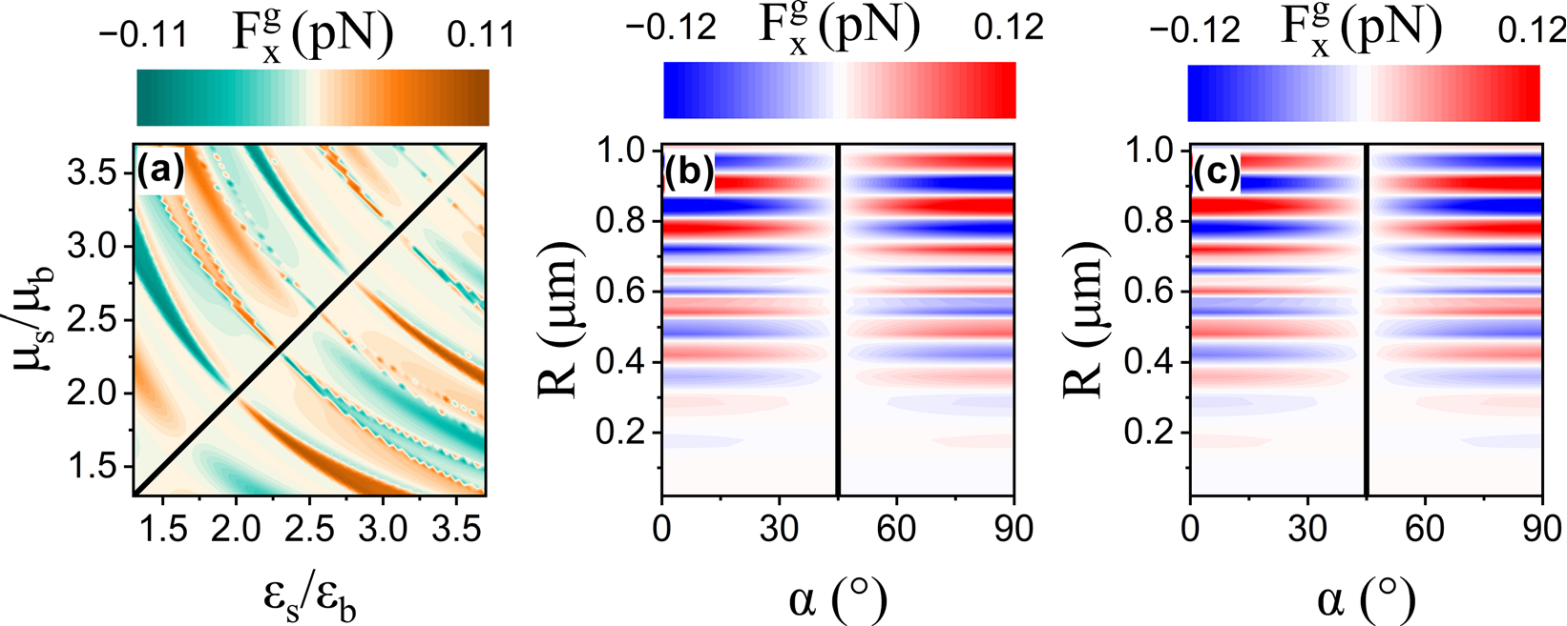
The longitudinal optical gradient force arising from the electromagnetic symmetry breaking of the particle. (a) Dependence of the gradient force on the particle’s relative permittivity *ɛ*_*s*_/*ɛ*_*b*_ and relative permeability *μ*_*s*_/*μ*_*b*_, with the particle immersed in vacuum. (b–d) Gradient force as a function of the polarization angle *α *and the particle radius *R* for two representative sets of electromagnetic parameters: (*ɛ*_*s*_, *μ*_*s*_) = (2.53, 1.0) in (b), (1.0, 2.53) in (c). The black solid lines indicate the conditions under which the optical gradient force disappears. All other parameters are the same as that of Figure 3c.

To gain deeper insight into the physical origin of gradient forces induced by electromagnetic symmetry breaking, Figure 4a shows that the gradient force is plotted as a function of the particle’s relative permittivity *ɛ*_*s*_ and permeability *μ*_*s*_, for a particle of 400 nm radius located at *x* = 400 nm. The black solid line indicates the condition *ɛ*_*s*_/*ɛ*_*b*_ = *μ*_*s*_/*μ*_*b*_, corresponding to identical electric and magnetic responses, where *ɛ*_*b*_ and *μ*_*b*_ are the background permittivity and permeability, respectively. As illustrated in Figure 4a, when the particle is immersed in vacuum, a gradient force emerges if *ɛ*_*s*_ ≠ *μ*_*s*_, indicating that electromagnetic symmetry has been broken. The gradient force also exhibits antisymmetry with respect to the solid black line, i.e., $F_{x}^{g}\left(\varepsilon_{s}/\varepsilon_{b},\mu_{s}/\mu_{b}\right)=-F_{x}^{g}\left(\mu_{s}/\mu_{b},\varepsilon_{s}/\varepsilon_{b}\right)$ , regardless of the particle size. Thus, for an isotropic and achiral particle of arbitrary size and composition, the condition *ɛ*_*s*_/*ɛ*_*b*_ ≠ *μ*_*s*_/*μ*_*b*_ leads to the emergence of a gradient force in an optical field that possesses antisymmetry in the electric and magnetic energy density gradients. When this antisymmetry between two components is broken, a gradient force can be generated even on particles with electromagnetic symmetry, as described in Figure S4 from the Supporting Information.

Furthermore, for particles with different *ɛ*_*s*_ and *μ*_*s*_, the dependence of the gradient force on both the polarization angle *α* and the particle radius *R* (ranging from the Rayleigh to the Mie scattering regime) is evaluated and presented in Figure 4b and c, respectively. No gradient force is observed across the entire parameter space for the symmetric case *ɛ*_*s*_ = *μ*_*s*_ = 2.53. The black curves denote the polarization angles at which the gradient force vanishes, corresponding to the absence of both electric and magnetic energy density gradients at *α* = 45° (consistent with Figure 2a and b). Intuitively, with a fixed polarization angle, variations in particle radius modulate both the magnitude and direction of the gradient force (consistent with Figure 3c). Moreover, for a fixed particle radius, the gradient force can be tuned by varying *α*, following a cosine cos(2*α*) dependence, independent of the particle’s size and composition. Notably, the gradient force reaches its maximum when *α* = 0° or 90°, implying that the maximum electric (or magnetic) energy density gradient is achieved at these angles. Similar trends are observed in Figure 4c for the case (*ɛ*_*s*_, *μ*_*s*_)=(1, 2.53). A comparison between Figure 4b and c confirms the antisymmetric property $F_{x}^{g}\left(2.53,1\right)=-F_{x}^{g}\left(1,2.53\right)$ . In contrast, the absence of a longitudinal gradient force for *ɛ*_*s*_ = *μ*_*s*_ = 2.53 can be attributed to the presence of electromagnetic symmetry. These results highlight the significant influence of both the polarization angle and particle radius on the magnitude and direction of the longitudinal gradient force. The ability to tune this force by adjusting the polarization angle provides new insights for optimizing optical manipulation.

## 3 Conclusions

While optical gradient forces have traditionally been regarded as a consequence of spatial variations in the intensity of the optical field, recent investigations have revealed that similar forces may also arise from gradients in more complex optical field quantities, such as the helicity density gradient [20], [21], [22], [23], [24] or the magneto-electric energy density gradient [25], albeit predominantly in systems involving chiral particles. In contrast to these earlier findings, the present work has demonstrated the existence of an unconventional gradient force that acts on isotropic, achiral spherical particles immersed in an interference field generated by two counter-propagating plane waves, wherein the total energy density gradient vanishes as a result of a polarization-tuned antisymmetry between its electric and magnetic components in the interference field. By establishing a general analytical expression for the gradient force exerted on particles of arbitrary size, it has been explicitly shown that the underlying physical origin of the observed force can be attributed to the particle-induced breaking of electromagnetic symmetry, which, in turn, couples to the antisymmetric structure of the energy density gradients inherent to the interfering field. Moreover, it has been found that the antisymmetry of the electric and magnetic energy density gradient distributions, can be effectively disrupted through appropriate adjustment of the illumination parameters, particularly the incident angle and polarization orientation, thereby enabling the generation of an optical gradient force even in the electric-magnetic symmetry of the particle. Of particular interest is the observation that the magnitude of the electric-magnetic symmetry-breaking-induced gradient force may reach its maximum value not under circular or linearly polarized light, but rather under elliptically polarized illumination at the specific position. Furthermore, through a systematic variation of the particle radius across the Rayleigh and Mie scattering regimes, it has been revealed that the contribution of the magnetic energy density gradient to the total optical force may, under certain conditions, surpass that arising from the electric component, thereby highlighting the critical role played by the magnetic energy density gradient in determining the overall force profile. These results not only deepen the fundamental understanding of light–matter interactions in structured fields but also offer novel opportunities for tailoring optical forces through the precise engineering of polarization states and material responses.

## Supporting Information

Numerical simulations of the energy density gradient under illumination by two plane waves with different incident angles are performed; the analytical derivation of optical forces on multipoles in two-wave interference is presented; the influence of the polarization state of the incident field on the optical gradient force is investigated; the breaking of antisymmetry in the electric and magnetic energy density gradients is analyzed.

## Supplementary Material

Supplementary Material Details
