# Supplementary material for: Anomalous optical gradient force induced by polarization-tuned antisymmetry in energy density gradient
Source: Nanophotonics. 2025 Sep 10;14(19):3125–32. doi: 10.1515/nanoph-2025-0223 (PMC12455292; doi:10.1515/nanoph-2025-0223)
Supplement: Supplementary file 1 — Supplementary Material Details [file j_nanoph-2025-0223_suppl_001.pdf]

Supporting Information for
Anomalous optical gradient force induced by polarization-tuned antisymmetry in energy density gradient

Lv Feng,[1,#] *Ziyi Su,*[1,#] *Ruohu Zhang*,[1] *Zhigang Li*,[1] *Bingjue Li*,[2] *and Guanghao Rui*[1,*]

[1]*Department of Optical Engineering, School of Electronic Science and Engineering, Southeast University, Nanjing, Jiangsu 211189, China*

[2]*School of Mechanical Engineering, Southeast University, Nanjing, Jiangsu 211189, China*

[#] Those authors contribute equally to the paper.

Corresponding author: [*]ghrui@seu.edu.cn

S1. The antisymmetry breaking in the energy density gradient of the interference field

Here, we consider the interference field consisting of two plane waves with incident angles (φ_1, φ_2)=(0°, 135°). When (α_1, α_2)=(0°, 90°) or (90°, 0°), the total energy density gradient in the x-direction vanishes, as in Figure S1(a), as its electric and magnetic components are both zero. Figures S1(b) and S1(c) demonstrate the antisymmetric breaking of the electric and magnetic energy density gradients, leading to the presence of a total energy density gradient. Consequently, by regulating the incident angle of the light and the orientation of polarization, the antisymmetry of the electric and magnetic energy density gradients is broken.

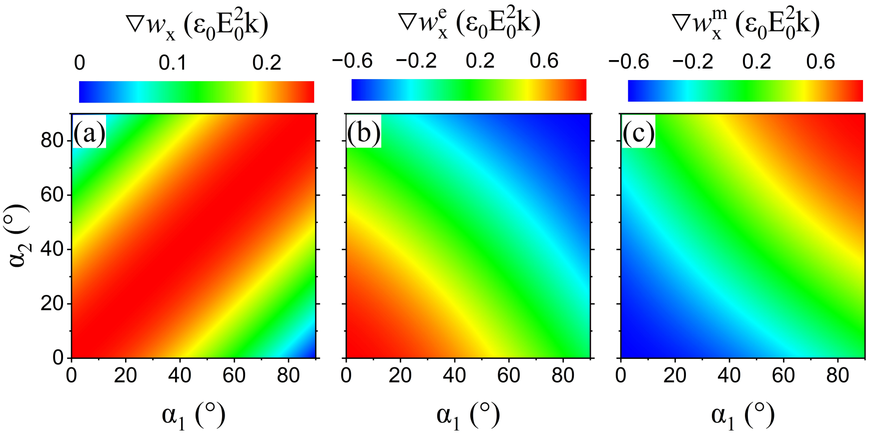

Figure S1: Numerical simulation of the anti-symmetry breaking in energy density gradient. Total energy density gradient (a) and its electric (b) and magnetic (c) components dependence on the polarization angles (α_1, α_2) in two-beam light field with incident angle (φ_1, φ_2) = (0°, 135°).

S2. Multipole expansion of optical force for multiple interferential plane-wave fields

An electromagnetic field consisting of multiple plane waves can be represented as

$$\mathbf{E} = \sum_{i=1}^{n} \mathbf{E}_i = \sum_{i=1}^{n} E_0 \boldsymbol{\mathcal{E}}_i e^{i\mathbf{k}_i\cdot\mathbf{r}}, \qquad \mathbf{B} = \sum_{i=1}^{n} \mathbf{B}_i = \sum_{i=1}^{n} B_0 \boldsymbol{\mathcal{B}}_i e^{i\mathbf{k}_i\cdot\mathbf{r}}, \tag{S1}$$

where E_0 is the amplitude of the plane wave, and $B_0 = E_0/c$, with c being the speed of light in vacuum. $k = 2\pi/\lambda$ and n denote the wave number and the number of plane waves, respectively. $\mathbf{k}_i$ denotes the wave vector of the ith plane wave with $\mathbf{k}_i = k\hat{\mathbf{k}}_i$, and $\boldsymbol{\mathcal{E}}_i$ and $\boldsymbol{\mathcal{B}}_i =$

$\hat{\mathbf{k}}_i \times \boldsymbol{\mathcal{E}}_i$ represent the complex amplitude vectors of the ith plane wave. Based on the multipole expansion theory, the time-averaged optical force is generally written as:[1,2]

$$\langle \mathbf{F} \rangle = \sum_{l=1}^{\infty} \langle \mathbf{F}_{\text{int}}^{(l)} \rangle + \sum_{l=1}^{\infty} \langle \mathbf{F}_{\text{rec}}^{(l)} \rangle , \tag{S2}$$

with

$$\langle \mathbf{F}_{\text{int}}^{(l)} \rangle = \langle \mathbf{F}_{\text{int}}^{\text{e}(l)} \rangle + \langle \mathbf{F}_{\text{int}}^{\text{m}(l)} \rangle, \qquad \langle \mathbf{F}_{\text{rec}}^{(l)} \rangle = \langle \mathbf{F}_{\text{rec}}^{\text{e}(l)} \rangle + \langle \mathbf{F}_{\text{rec}}^{\text{m}(l)} \rangle + \langle \mathbf{F}_{\text{rec}}^{\text{x}(l)} \rangle, \tag{S3}$$

where l denotes the multipole order, which corresponds to dipole, quadrupole, and octupole when l = 1, 2, 3, respectively. $\mathbf{F}_{\text{int}}^{(l)}$ expresses the intercepting force, which can be simply understood as the interaction between the electromagnetic multipole and the incident field. The $\mathbf{F}_{\text{rec}}^{(l)}$ is the recoil force that indicates the coupling between multipoles of the same type (electric or magnetic) of neighboring orders, and between electric and magnetic multipoles of the same order. To simplify the formula iteration, we set $E_0 = B_0 = k = c = \omega = \varepsilon_0 = \mu_0 = 1$, to derive the formula in the dimensionless case such that its optical force is in units of $\varepsilon_b E_0^2/k^2$, where ε_b is the dielectric constant of the background.

The interception and recoil forces are given as:[2]

$$\begin{aligned}
\langle \mathbf{F}_{\text{int}}^{\text{e}(l)} \rangle &= -u_l^{(1)} \sum_{i,j} \{\text{Im}[a_l \mathbf{Y}_{l,ij}^{(1)}]\}, \\
\langle \mathbf{F}_{\text{int}}^{\text{m}(l)} \rangle &= -u_l^{(1)} \sum_{i,j} \{\text{Im}[b_l \mathbf{Y}_{l,ij}^{(2)}]\}, \\
\langle \mathbf{F}_{\text{rec}}^{\text{e}(l)} \rangle &= -u_l^{(2)} \sum_{i,j} \text{Im}[a_l^* a_{l+1} \mathbf{Y}_{l,ij}^{(3)}]\}, \\
\langle \mathbf{F}_{\text{rec}}^{\text{m}(l)} \rangle &= -u_l^{(2)} \sum_{i,j} \text{Im}[b_l^* b_{l+1} \mathbf{Y}_{l,ij}^{(4)}]\}, \\
\langle \mathbf{F}_{\text{rec}}^{\text{x}(l)} \rangle &= u_l^{(3)} \sum_{i,j} \text{Re}[a_l^* b_l \mathbf{Y}_{l,ij}^{(5)}],
\end{aligned} \tag{S4}$$

where

$$u_l^{(1)} = \frac{\pi(2l+1)}{l(l+1)}, \quad u_l^{(2)} = \frac{\pi}{2(l+1)^2}, \quad u_l^{(3)} = \frac{\pi(2l+1)}{2l^2(l+1)^2}, \tag{S5}$$

which ij refers to the ij-th plane wave, a_l and b_l are electric and magnetic Mie coefficients determined by the character of the particles. Vector **Y**, which is related to and vectors **Z** and **S**, is defined as:

$$\begin{aligned}
\mathbf{Y}_{l,ij}^{(1)} &= Q_{l,ij}^{(1)}\mathbf{Z}_{\mathrm{ee},ij}^{(1)} - Q_{l,ij}^{(2)}\mathbf{Z}_{\mathrm{mm},ij}^{(1)}, \\
\mathbf{Y}_{l,ij}^{(2)} &= Q_{l,ij}^{(1)}\mathbf{Z}_{\mathrm{mm},ij}^{(1)} - Q_{l,ij}^{(2)}\mathbf{Z}_{\mathrm{ee},ij}^{(1)}, \\
\mathbf{Y}_{l,ij}^{(3)} &= R_{l,ij}^{(1)}\mathbf{Z}_{\mathrm{ee},ij}^{(1)*} - R_{l,ij}^{(2)}\mathbf{Z}_{\mathrm{mm},ij}^{(1)*} - 4iR_{l,ij}^{(3)}\mathbf{S}_{\mathrm{em},ij}^{(1)*} + R_{l,ij}^{(4)}\mathbf{Z}_{\mathrm{ee},ij}^{(1)} - R_{l,ij}^{(5)}\mathbf{Z}_{\mathrm{mm},ij}^{(1)} + 4iR_{l,ij}^{(6)}\mathbf{S}_{\mathrm{em},ij}^{(1)}, \\
\mathbf{Y}_{l,ij}^{(4)} &= R_{l,ij}^{(1)}\mathbf{Z}_{\mathrm{mm},ij}^{(1)*} - R_{l,ij}^{(2)}\mathbf{Z}_{\mathrm{ee},ij}^{(1)*} - 4iR_{l,ij}^{(3)}\mathbf{S}_{\mathrm{em},ij}^{(1)} + R_{l,ij}^{(4)}\mathbf{Z}_{\mathrm{mm},ij}^{(1)} - R_{l,ij}^{(5)}\mathbf{Z}_{\mathrm{ee},ij}^{(1)} + 4iR_{l,ij}^{(6)}\mathbf{S}_{\mathrm{em},ij}^{(1)*}, \\
\mathbf{Y}_{l,ij}^{(5)} &= iR_{l,ij}^{(4)}[\mathbf{Z}_{\mathrm{ee},ij}^{(1)} - \mathbf{Z}_{\mathrm{mm},ij}^{(1)*}] + iR_{l,ij}^{(5)}[\mathbf{Z}_{\mathrm{ee},ij}^{(1)*} - \mathbf{Z}_{\mathrm{mm},ij}^{(1)}] - 4R_{l,ij}^{(7)}\mathbf{S}_{\mathrm{em},ij}^{(1)*} - 4R_{l,ij}^{(6)}\mathbf{S}_{\mathrm{em},ij}^{(1)},
\end{aligned} \tag{S6}$$

where $\mathbf{Z}_{\mathrm{ee},ij}^{(1)}$, $\mathbf{Z}_{\mathrm{mm},ij}^{(1)}$, $\mathbf{Z}_{\mathrm{em},ij}^{(1)}$, and $\mathbf{Z}_{\mathrm{me},ij}^{(1)}$ are field quantities depending on the incident field, given by:

$$\begin{aligned}
\mathbf{Z}_{\mathrm{ee},ij}^{(1)} &= \frac{1}{2}[\nabla \mathrm{D}_{\mathrm{ee},ij}^{(1)} - \nabla\times\mathbf{S}_{\mathrm{ee},ij}^{(1)} - 2i\,\mathrm{Re}\,\mathbf{S}_{\mathrm{em},ij}^{(1)}], \\
\mathbf{Z}_{\mathrm{mm},ij}^{(1)} &= \frac{1}{2}[\nabla \mathrm{D}_{\mathrm{mm},ij}^{(1)} - \nabla\times\mathbf{S}_{\mathrm{mm},ij}^{(1)} - 2i\,\mathrm{Re}\,\mathbf{S}_{\mathrm{em},ij}^{(1)}],
\end{aligned} \tag{S7}$$

where the field quantities for each pair of plane waves are given by:

$$\begin{aligned}
\mathbf{S}_{\mathrm{em},ij}^{(1)} &= (\boldsymbol{\mathcal{E}}_i \times \boldsymbol{\mathcal{B}}_j^*)e^{i(\mathbf{k}_i-\mathbf{k}_j)\cdot\mathbf{r}}, \\
\mathbf{S}_{\mathrm{ee},ij}^{(1)} &= (\boldsymbol{\mathcal{E}}_i \times \boldsymbol{\mathcal{E}}_j^*)e^{i(\mathbf{k}_i-\mathbf{k}_j)\cdot\mathbf{r}}, \\
\mathbf{S}_{\mathrm{mm},ij}^{(1)} &= (\boldsymbol{\mathcal{B}}_i \times \boldsymbol{\mathcal{B}}_j^*)e^{i(\mathbf{k}_i-\mathbf{k}_j)\cdot\mathbf{r}}, \\
\nabla \mathrm{D}_{\mathrm{ee},ij}^{(1)} &= i(\mathbf{k}_i - \mathbf{k}_j)(\boldsymbol{\mathcal{E}}_i \cdot \boldsymbol{\mathcal{E}}_j^*)e^{i(\mathbf{k}_i-\mathbf{k}_j)\cdot\mathbf{r}}, \\
\nabla \mathrm{D}_{\mathrm{mm},ij}^{(1)} &= i(\mathbf{k}_i - \mathbf{k}_j)(\boldsymbol{\mathcal{B}}_i \cdot \boldsymbol{\mathcal{B}}_j^*)e^{i(\mathbf{k}_i-\mathbf{k}_j)\cdot\mathbf{r}}, \\
\nabla\times\mathbf{S}_{\mathrm{ee},ij}^{(1)} &= i(\mathbf{k}_i - \mathbf{k}_j)\times(\boldsymbol{\mathcal{E}}_i \times \boldsymbol{\mathcal{E}}_j^*)e^{i(\mathbf{k}_i-\mathbf{k}_j)\cdot\mathbf{r}}, \\
\nabla\times\mathbf{S}_{\mathrm{mm},ij}^{(1)} &= i(\mathbf{k}_i - \mathbf{k}_j)\times(\boldsymbol{\mathcal{B}}_i \times \boldsymbol{\mathcal{B}}_j^*)e^{i(\mathbf{k}_i-\mathbf{k}_j)\cdot\mathbf{r}},
\end{aligned} \tag{S8}$$

The coefficients $\mathcal{Q}$ and $\mathcal{R}$ are defined as follows:

$$
\begin{aligned}
Q_{l,ij}^{(1)} &= \sum_{m=1}^{l}{}^{(2)} m(2l+1-m)(2l+1-2m)P_{l-m}(x_{ij}), \\
Q_{l,ij}^{(2)} &= \sum_{m=2}^{l}{}^{(2)} m(2l+1-m)(2l+1-2m)P_{l-m}(x_{ij}), \\
R_{l,ij}^{(1)} &= \sum_{m=1}^{l}{}^{(2)} (m+1)(2l+2-m)(2l+1-2m)[2(m+1)l-(m^2-m-4)]P_{l-m}(x_{ij}), \\
R_{l,ij}^{(2)} &= \sum_{m=2}^{l}{}^{(2)} m(m+2)(2l+1-m)(2l+1-2m)(2l+3-m)P_{l-m}(x_{ij}), \\
R_{l,ij}^{(3)} &= \sum_{m=1}^{l}{}^{(2)} (m+1)(2l+2-m)(2l+1-2m)P_{l-m}(x_{ij}), \\
R_{l,ij}^{(4)} &= \sum_{m=2}^{l}{}^{(2)} (2l+1-m)(2l+1-2m)[2m^2l-m(m+1)(m-2)]P_{l-m}(x_{ij}), \\
R_{l,ij}^{(5)} &= \sum_{m=1}^{l}{}^{(2)} (m+1)(m-1)(2l-m)(2l+2-m)(2l+1-2m)P_{l-m}(x_{ij}), \\
R_{l,ij}^{(6)} &= \sum_{m=2}^{l}{}^{(2)} m(2l+1-m)(2l+1-2m)P_{l-m}(x_{ij}), \\
R_{l,ij}^{(7)} &= \sum_{m=1}^{l}{}^{(2)} (2l+1-2m)[2l^2-2(m-1)l+m^2-m]P_{l-m}(x_{ij}),
\end{aligned}
\tag{S9}
$$

where $P_l(x)$ is a Legendre polynomial, and m is an odd (even) positive integer satisfying $m \le l$ in the summation $\sum_{m=1}^{l}{}^{(2)}$ ($\sum_{m=2}^{l}{}^{(2)}$).

S3. Formulations of optical forces on multipoles in two-wave interference

The previous subsection focused on describing the total optical force exerted on an achiral spherical particle by an optical field superimposed by arbitrarily polarized plane waves. Next, we derive, based on the theory of the previous section, the optical force exerted on an isotropic achiral spherical particle immersed in an optical field composed of two plane waves as discussed in the main text. Plane waves with arbitrarily polarization and all their wave vectors located on the *xoy* plane. Therefore, the polar angle of each plane wave is $\theta_j = \pi/2$, wave vector $\widehat{\mathbf{k}}_j = cos\varphi_j\,\hat{\mathbf{x}} + sin\varphi_j\,\hat{\mathbf{y}}$, where φ_j denotes the azimuth of the j-th plane wave. In this paper, the electric and magnetic fields in the Cartesian coordinate system of the j-th plane wave are given by

$$\mathbf{E}_j = (-q_j\ sin\varphi_j \hat{\boldsymbol{x}} + q_j\ cos\varphi_j \hat{\boldsymbol{y}} - p_j\ \hat{\boldsymbol{z}}) e^{i(cos\varphi_j x + sin\varphi_j y)},$$
$$\mathbf{B}_j = (-p_j\ sin\varphi_j \hat{\boldsymbol{x}} + p_j\ cos\varphi_j \hat{\boldsymbol{y}} + q_j\ \hat{\boldsymbol{z}}) e^{i(cos\varphi_j x + sin\varphi_j y)}, \quad \text{(S10)}$$

with

$$\boldsymbol{\mathcal{E}}_j = -q_j\ sin\varphi_j \hat{\boldsymbol{x}} + q_j\ cos\varphi_j \hat{\boldsymbol{y}} - p_j\ \hat{\boldsymbol{z}},$$
$$\boldsymbol{\mathcal{B}}_j = -p_j\ sin\varphi_j \hat{\boldsymbol{x}} + p_j\ cos\varphi_j \hat{\boldsymbol{y}} + q_j\ \hat{\boldsymbol{z}}, \quad \text{(S11)}$$

Substituting Eq. (S10) into Eq. (S8), with $\varphi_1 = 0$ and $\varphi_2 = \pi$, and the x-component of the relevant field quantity for a pair of plane waves can be obtained by simple algebra, viz.

$$
\begin{aligned}
&(\nabla \times \mathbf{S}^{(1)}_{\mathrm{ee},ij})_x = 0, && (\nabla \times \mathbf{S}^{(1)}_{\mathrm{mm},ij})_x = 0,\\
&(\mathbf{S}^{(1)}_{\mathrm{em},11})_x = p_1 p_1^* + q_1 q_1^*, && (\mathbf{S}^{(1)}_{\mathrm{em},22})_x = -p_2 p_2^* - q_2 q_2^*,\\
&(\mathbf{S}^{(1)}_{\mathrm{em},12})_x = (-p_1 p_2^* + q_1 q_2^*) e^{2ix}, && (\mathbf{S}^{(1)}_{\mathrm{em},21})_x = (p_2 p_1^* - q_2 q_1^*) e^{-2ix},\\
&(\nabla \mathrm{D}^{(1)}_{\mathrm{ee},12})_x = 2i(p_1 p_2^* - q_1 q_2^*) e^{2ix}, && (\nabla \mathrm{D}^{(1)}_{\mathrm{ee},21})_x = -2i(p_2 p_1^* - q_2 q_1^*) e^{-2ix},\\
&(\nabla \mathrm{D}^{(1)}_{\mathrm{mm},12})_x = 2i(-p_1 p_2^* + q_1 q_2^*) e^{2ix}, && (\nabla \mathrm{D}^{(1)}_{\mathrm{mm},21})_x = 2i(p_2 p_1^* - q_2 q_1^*) e^{-2ix}.
\end{aligned}
\quad \text{(S12)}
$$

When i=j, with x_{11}=x_{22}=1 and $|p_j|^2 + |q_j|^2 = 1$, then $\mathbf{S}^{(1)}_{\mathrm{em},11} + \mathbf{S}^{(1)}_{\mathrm{em},22} = 0$. In the main manuscript, we assume that $p_j = cos(\alpha_j)$ and $q_j = sin(\alpha_j) e^{i\beta_j}$, where α_j describes the angle between the polarization direction of the electric field and the z-axis, and β_j indicates the phase difference between the y and z components of the electric field. When $i \neq j$ with x_{12}=x_{21}=−1, then the electric and magnetic parts of the energy density gradient are indicated as, respectively,

$$\nabla w_e = \frac{1}{4} \nabla \mathrm{D}^{(1)}_{\mathrm{ee}}, \qquad \nabla w_m = \frac{1}{4} \nabla \mathrm{D}^{(1)}_{\mathrm{mm}}, \quad \text{(S13)}$$

where

$$\nabla \mathrm{D}^{(1)}_{\mathrm{ee}} = 2\,\mathrm{Re}[2i(p_1 p_2^* - q_1 q_2^*) e^{2ix}]\, \hat{\boldsymbol{x}},$$
$$\nabla \mathrm{D}^{(1)}_{\mathrm{mm}} = 2\,\mathrm{Re}[2i(-p_1 p_2^* + q_1 q_2^*) e^{2ix}]\, \hat{\boldsymbol{x}}, \quad \text{(S14)}$$

and

$$\sum_{i,j} \mathrm{Im}[\mathbf{S}^{(1)}_{\mathrm{em},ij}] = \mathrm{Im}[\mathbf{S}^{(1)}_{\mathrm{em},11} + \mathbf{S}^{(1)}_{\mathrm{em},12} + \mathbf{S}^{(1)}_{\mathrm{em},21} + \mathbf{S}^{(1)}_{\mathrm{em},22}]$$

$$= -\frac{1}{4}[\nabla \mathrm{D}^{(1)}_{\mathrm{mm}} - \nabla \mathrm{D}^{(1)}_{\mathrm{ee}}] = [\nabla w_e - \nabla w_m],$$

$$\sum_{i,j} \mathbf{Z}^{(1)}_{\mathrm{ee},ij} = \frac{1}{2}\nabla \mathrm{D}^{(1)}_{\mathrm{ee}} = 2\nabla w_e, \tag{S15}$$

$$\sum_{i,j} \mathbf{Z}^{(1)}_{\mathrm{mm},ij} = \frac{1}{2}\nabla \mathrm{D}^{(1)}_{\mathrm{mm}} = 2\nabla w_m,$$

$$\sum_{i,j} \mathrm{Re}[\mathbf{S}^{(1)}_{\mathrm{em},ij}] = 0.$$

The vector **Y** is derived as

$$\begin{aligned}
\sum_{i,j} \mathbf{Y}^{(1)}_{l,ij} &= Q^{(1)}_{l,ij} 2\nabla w_e - Q^{(2)}_{l,ij} 2\nabla w_m, \\
\sum_{i,j} \mathbf{Y}^{(2)}_{l,ij} &= Q^{(1)}_{l,ij} 2\nabla w_m - Q^{(2)}_{l,ij} 2\nabla w_e, \\
\sum_{i,j} \mathbf{Y}^{(3)}_{l,ij} &= R^{(1)}_{l,ij} 2\nabla w_e - R^{(2)}_{l,ij} 2\nabla w_m - 4R^{(3)}_{l,ij}[\nabla w_e - \nabla w_m] \\
&\quad + R^{(4)}_{l,ij} 2\nabla w_e - R^{(5)}_{l,ij} 2\nabla w_m - 4R^{(6)}_{l,ij}[\nabla w_e - \nabla w_m], \\
\sum_{i,j} \mathbf{Y}^{(4)}_{l,ij} &= R^{(1)}_{l,ij} 2\nabla w_m - R^{(2)}_{l,ij} 2\nabla w_e + 4R^{(3)}_{l,ij}[\nabla w_e - \nabla w_m] \\
&\quad + R^{(4)}_{l,ij} 2\nabla w_m - R^{(5)}_{l,ij} 2\nabla w_e + 4R^{(6)}_{l,ij}[\nabla w_e - \nabla w_m], \\
\sum_{i,j} \mathbf{Y}^{(5)}_{l,ij} &= iR^{(4)}_{l,ij}[2\nabla w_e - 2\nabla w_m] + iR^{(5)}_{l,ij}[2\nabla w_e - 2\nabla w_m] \\
&\quad + 4iR^{(7)}_{l,ij}[\nabla w_e - \nabla w_m] - 4iR^{(6)}_{l,ij}[\nabla w_e - \nabla w_m],
\end{aligned} \tag{S16}$$

and the analytical expression for the optical force is finally obtained by

$$\begin{aligned}
\mathbf{F}^{(l)}_{\mathrm{int}} &= (-1)^l \frac{(2l+1)\pi}{2} \mathrm{Im}[(\zeta_l a_l + \xi_l b_l)\nabla w_e + (\xi_l a_l + \zeta_l b_l)\nabla w_m], \\
\mathbf{F}^{(l)}_{\mathrm{rec}} &= (-1)^l \chi_l \pi\, \mathrm{Im}[(\gamma_l a_l^* a_{l+1} + \eta_l b_l^* b_{l+1} + 2\upsilon_l a_l^* b_l)\nabla w_e \\
&\quad + (\eta_l a_l^* a_{l+1} + \gamma_l b_l^* b_{l+1} - 2\upsilon_l a_l^* b_l)\nabla w_m],
\end{aligned} \tag{S17}$$

with

$$\begin{aligned}
\zeta_l &= l^2 + l + 2, & \xi_l &= l^2 + l - 2, \\
\gamma_l &= l^2 + 2l + 3, & \eta_l &= l^2 + 2l - 1, \\
\chi_l &= \frac{l(l+2)}{l+1}, & \upsilon_l &= \frac{(2l+1)}{l^2(l+2)}.
\end{aligned} \tag{S18}$$

Equation (S17) demonstrates that the optical force in a standing wave originates from the gradient force, proportional to the gradient of the electric and magnetic energy densities.

S4. Optical gradient force in two plane waves with same polarization

To understand intuitively the extraordinary polarization-induced anti-symmetric mechanism and the longitudinal gradient force, we consider a two-wave interference field with plane waves all having the same polarization state. Figure S2 illustrates the variation of optical gradient force with the polarization angle α and the phase difference β. Subsequently, the amplitude and sign of the optical gradient force can be tunable by the polarization angle α ranging from 0 ° to 90 ° independent of the phase difference β, exhibiting a $\cos(2\alpha)$ dependence. Notably, the optical gradient force can reach a maximum in amplitude at α=0 or 90° and also disappears at α= 45°, which is attributed to the absence of both electric and magnetic energy density gradients.

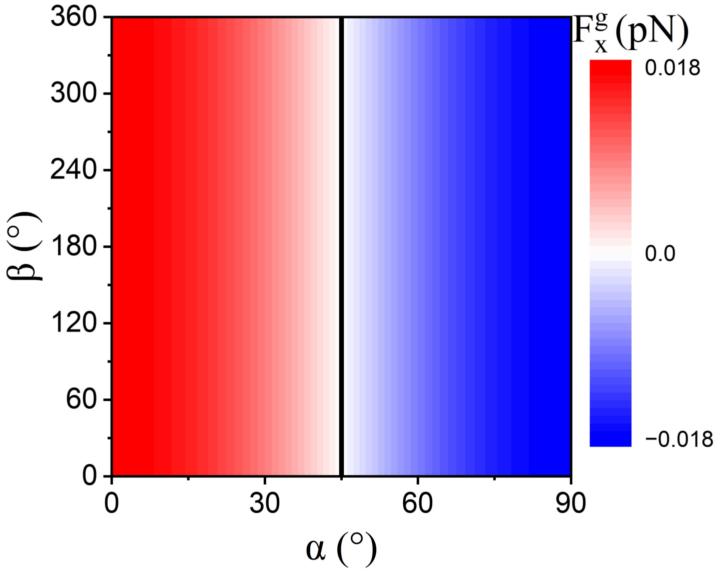

Figure S2: The longitudinal gradient force in two plane waves with same polarization. The optical gradient force versus the polarization angle α and the phase difference β. The black solid line denote the vanishing of the optical gradient force. The incident wavelength is 532 nm. The particle has a radius of 400 nm and is located at x = 400 nm, with a relative permittivity of 2.53 and relative permeability of 1.0.

S5. Optical gradient force in two plane waves with different polarization

For two incident plane waves having different polarization states, first, with fixed the phase difference β_1=β_2=0, Figure S3(a) demonstrates the variation of the optical gradient force as a function of the polarization angles α_1 and α_2. When the two polarization angles are complementary, i.e., $\alpha_1 + \alpha_2 = \pi/2$, Neither the electric nor the magnetic energy density gradient is present, resulting in the disappearance of the optical gradient force as shown by the black lines in Fig. S3(a). In addition, Figure S3(a) demonstrates a hidden symmetry relationship, where the optical gradient force exhibits anti-symmetry with respect to the black line, i.e. $F_x^g(\alpha_1, \alpha_2) =- F_x^g(\frac{\pi}{2} - \alpha_2, \frac{\pi}{2} - \alpha_1)$. Secondly, fixing $\alpha_1 = \alpha_2 = \pi/4$, Figure S3(b) exhibits the gradient force as a function of the phase difference β_1 and β_2. It is obvious that there exists F_x^g=0, when β_1=β_2 or (β_1, β_2)=(0, 2π)[(2π, 0)] is satisfied. Notably, when considering elliptical polarization, the optical gradient force is an order of magnitude larger in amplitude than that for linear polarization. The two white lines in Figure S3 are denoted as $\beta_1 - \beta_2 =- 91°$ and $\beta_1 - \beta_2 = 269°$, respectively, signifying that the gradient force reaches its maximum value. Consequently, by tuning the polarization state, an effective method is provided for controlling the amplitude and sign of the electric and magnetic energy density gradients.

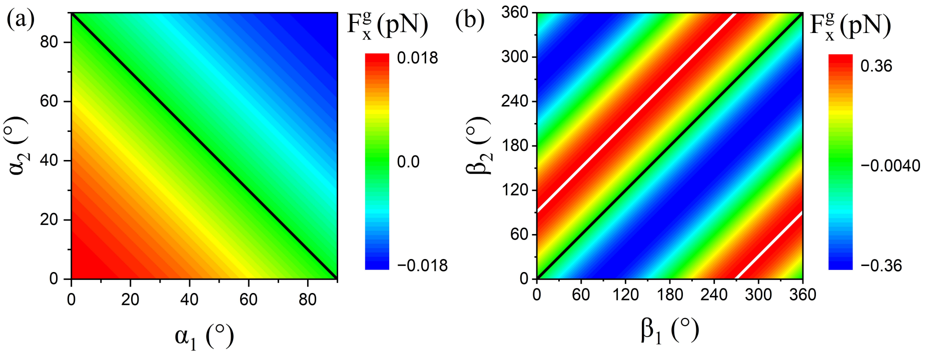

Figure S3: Longitudinal optical gradient force in two plane waves with different polarization states. (a) Optical gradient force as a function of the polarization angles α_1 and α_2, with the phase difference fixed at β_1= β_2=0°. (b) Optical gradient force as a function of the phase differences β_1 and β_2, with the polarization angles fixed at α_1= α_2=45°. The black solid lines indicate the conditions under which the optical gradient force vanishes, while the

white lines represent the locations where the gradient force reaches its maximum value. All other parameters are the same as that of Figure S2.

S6. Optical gradient force acting on the particle with the electromagnetic symmetry

Based on the dipole approximation, Figure S4 demonstrates the gradient force as a function of the relative permittivity $\varepsilon_s/\varepsilon_b$ and relative permeability μ_s/μ_b of the particle immersed in two-beam light field with incident angle (φ_1, φ_2)=(0°, 135°), the polarization angle (α_1, α_2)=(0°, 45°), and the phase difference β_1= β_2=0°, where the particle has a radius of 50 nm, and is located at x = 400 nm. Notably, the optical gradient force still exists even if the particles have the same electric and magnetic responses, due to the breaking of the antisymmetry between the electric and magnetic components of the energy density gradient, as shown in Figure S1. In other words, when the anti-symmetry of the electric and magnetic energy density gradient is broken, the gradient force is found to arise on the particles with electromagnetic symmetry.

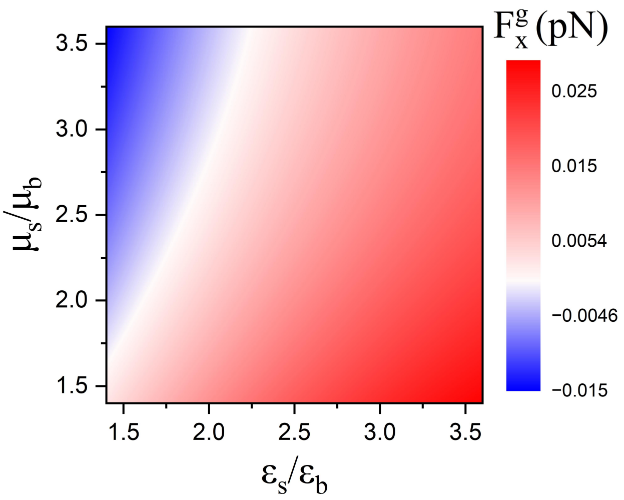

Figure S4: The optical gradient force versus the relative permittivity $\varepsilon_s/\varepsilon_b$ and relative permeability μ_s/μ_b of the particle immersed in two-beam light field with incident angle (φ_1, φ_2)=(0°, 135°), the polarization angle (α_1, α_2)=(0°, 45°), and the phase difference β_1= β_2=0°. The particle has a radius of 50 nm and is placed in vacuum.

References

[1] X. N. Yu, Y. K. Jiang, S. Y. Liu, and Z. F. Lin, "Approach to fully decomposing an optical force into conservative and nonconservative components," *Phys. Rev. A*, vol. 100, no. 3, p. 033821, 2019.

[2] H. Zheng, X. Li, Y. Jiang, J. Ng, Z. Lin, and H. Chen, "General formulations for computing the optical gradient and scattering forces on a spherical chiral particle immersed in generic monochromatic optical fields," *Phys. Rev. A*, vol. 101, no. 5, p. 053830, 2020.
